# Supplementary material for: Assessment of Diagnostic Specificity of Anti-SARS-CoV-2 Antibody Tests and Their Application for Monitoring of Seroconversion and Stability of Antiviral Antibody Response in Healthcare Workers in Moscow
Source: Microorganisms. 2022 Feb 12;10(2):429. doi: 10.3390/microorganisms10020429 (PMC8874386; doi:10.3390/microorganisms10020429)
Supplement: Supplementary file 1 [file microorganisms-10-00429-s001.zip › microorganisms-1516702-supplementary.pdf]

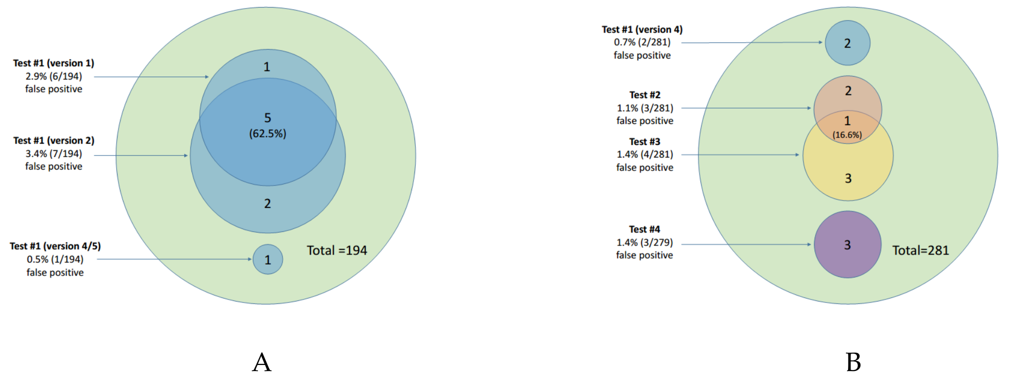

Figure S1. Venn diagrams showing distribution of false-negative results between generations 1, 2 and 4/5 of test #1 (A) and between tests #1, #2, #3, and #4 (B). Numbers in small circles represent the number of reactive sera; the proportions of samples with concordant reactive results are shown in percent; total number represent the number of archive samples tested.

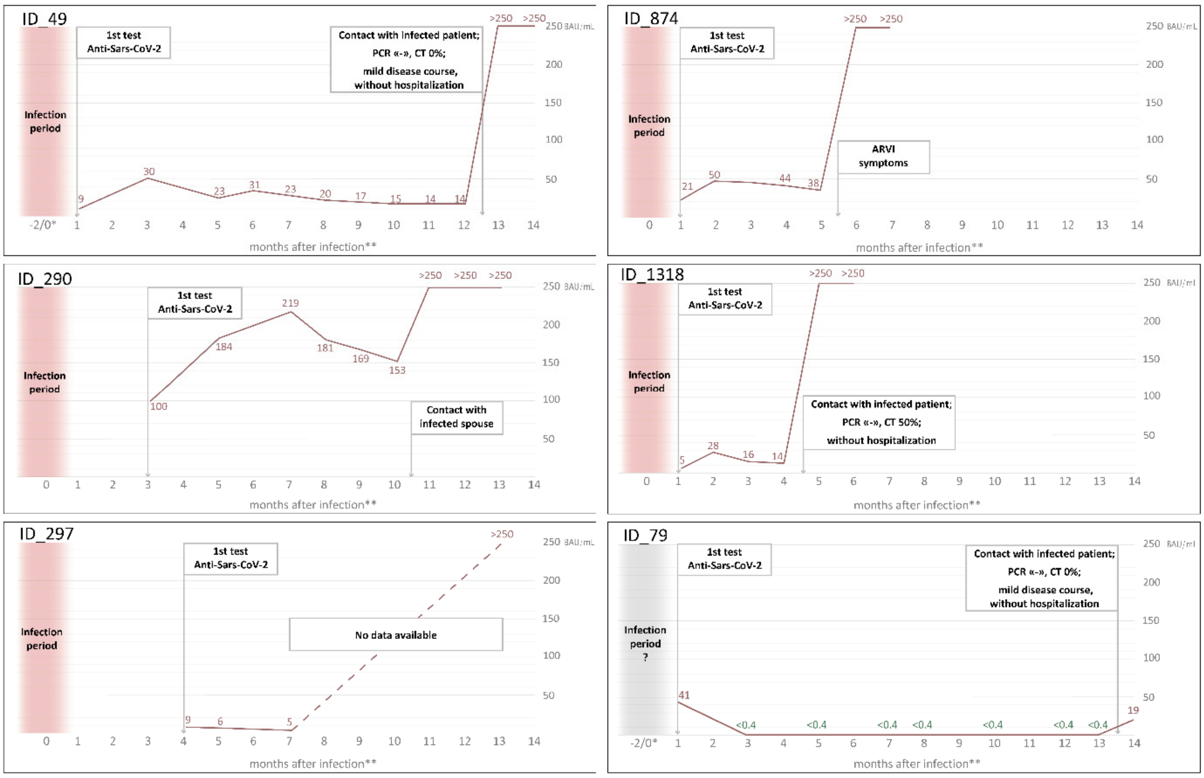

Figure S2. Cases of anti-S antibody boosting during follow up period. Note: In case of participant ID_79 the error at the initial testing cannot be excluded. Thus, this case of possible antibody boosting is uncertain and shown in gray. * The date of infection is unknown (asymptomatic infection), however, given the time when the COVID-19 pandemic began in Moscow, it cannot exceed two months. ** The date of infection was set according to data reported in questionnaire. CT %, the proportion of lung damage by CT diagnostics. COI, cut-off index. BAU/mL, binding antibody units per mL.

According questionnaire data reported by participants, all 5 cases had mild or asymptomatic primary SARS-CoV-2. In another asymptomatic case (ID_79, Figure 7 and Supplementary Table S3), we observed the reappearance of anti-S antibodies at concentration 19 BAU/ml, which may also indicate re-exposure to the virus and antibody booster, or a sampling error that led to the false-positive result in initial testing. It should be noted that 2 months after the reappearance of detectable antibodies, against the background of recorded contact with an infected person and two-dose vaccination, this participant had concentration of anti-S antibodies only as high as 17.1 BAU/ml.

In 5 out of 6 cases the antibody booster was observed in participants with background concentrations <40 BAU/ml, and in one case, a sharp increase occurred from the background level of 150 BAU/ml. In 3 out of 6 cases presented (ID_49, ID_1318, and ID_79, all healthcare workers), COVID-19 has been diagnosed during the period of an increase in the antibody concentrations. All these cases received outpatient treatment. Epidemiological and clinical data during the period of antibody boosting for other three cases (all administrative staff) are limited. One case (ID_290) reported a contact with infected spouse, the second (ID_297) came after a long break (6 months) between tests and did not provide any information, and the third retrospectively reported the presence of signs of ARVI that did not raise suspicions of COVID-19.

Table S1. Serum samples of healthcare workers used in the study.

Used in the study stage	Cohort/sub-cohort		Nn of participants	Nn of sera	Criteria of inclusion	Assessed with test system #
Step 1, Task 1.1 Performance of anti-SARS-CoV-2 tests	Conditionally healthy healthcare workers from Belgorod region (*n*=194) and Kaliningrad region (*n*=87) of Russia Time of collection 2018 and 2019 respectively		281	281	No exposition to SARS-CoV-2 (pre-epidemics)	1,2,3, 4,5
Step 2, Task 2.1 Anti-SARS-CoV-2 Prevalence rates at initial screening done at different stages of COVID-19 pandemic	Healthcare workers: employees of RMANPO (Moscow, Russia) included medical personnel, teachers, students, technical and administrative staff	All interested employees	527	527	Have not been applied	3,4
Step 2, Task 2.2 Duration of reactivity for IgM antibodies to SARS-CoV-2		Sub-cohort 1	63	374 (2 to 19 per participant)	Participants who had ≥ 4 months between the first positive IgM/IgG test (not from the self-reported date of infection) and the last testing (126 to 396 days, M = 228.8; SD = 75.12)	4,7
Step 2, Task 2.3 Monthly rates of anti-SARS-CoV-2 seroconversion		Sub-cohort 2	100	986 (3 to 22 per participant)	Participants who had the first test done before June 2020 and whose anti-SARS-CoV-2 antibody status was known by the end of the study (June 2021)	4
Step 2, Task 2.4 Changes in anti-S antibody concentrations		Sub-cohort 3	56	279 (2 to 10 per participant)	Participants who had ≥ 6 months between the first positive IgG test and the last testing (159 to 411 days, M = 262, SD = 75.8). Did not include participants with post-vaccination antibodies	6
Step 1, Task 1.3 Correlation of tests		Sub-cohort 4	34	160 (2 to 8 per participant	Participants who had ≥ 5 months between the first positive IgG test and the last testing (at the time of the analysis in April 2021)	3,5,6

Table S2. Anti-SARS-CoV-2 antibody testing results for dilutions of research reagent for anti-SARS-CoV-2 antibodies (NIBSC code 20/130)

Dilution	Test (manufacturer)					
	DS IFA-ANTI-SARS-CoV-2, version 4/5 (NPO "Diagnostic Systems", Russia)		SARS-CoV-2-IgG-IFA (National Research Center for Hematology, Moscow, Russia)		SARS-CoV-2-IgG-IFA-BEST (Vector-Best, Novosibirsk, Russia)	
1:500	OD*	Result	OD	Result	OD	Result
1:1,000	3.778	reactive	0.769	reactive	1.374	reactive
1:1,200	2.436	reactive	0.598	reactive	0.540	reactive
1:1,400	n.t.	n.t.	n.t.	n.t.	0.275	non-reactive
1:1,600	n.t.	n.t.	n.t.	n.t.	0.184	non-reactive
1:2,000	n.t.	n.t.	n.t.	n.t.	0.164	non-reactive
1:4,000	0.966	reactive	0.299	non-reactive	n.t.	n.t.
1:6,000	0.451	reactive	0.193	non-reactive	n.t.	n.t.
Calculated endpoint titer	1:5,558		1:1,428		1:1,188	

* mean for three replicates. n.t. – not tested

Table S3. Inconsistent individual serological profiles observed in three study participants.

Patient	Test	Observation period, months (times testing in month)													
		1	3	5	6	7(1)	7(2)	8(1)	8(2)	10	11	12	13(1)	13(2)	14
ID_49	#4 (anti-N & S-RBD IgM)	-	-	-	-	-	-	-	-	-	-	-	-	+	+
	#3 (anti-S IgG)	+	+	+	+	-	+	g.z.	-	+	+	+	+	+	+
	#5 (total anti-N)	+	+	+	-	-	-	-	-	-	-	-	-	+	+
	#6 (total anti-S) BAU/ml	8.95	29.78	22.53	31.02	27.14	23.22	19.94	19.85	15.74	13.85	13.77	18.37	>250	>250

Patient	Test	Observation period, months									
		1	2	3	5	7	8	10	12	13	14
ID_79	#4 (anti-N & S-RBD IgM)	+	-	-	-	-	-	-	-	-	+
	#3 (anti-S IgG)	+	-	-	-	-	-	-	-	-	+
	#5 (total anti-N)	+	-	-	-	-	-	-	-	-	+
	#6 (total anti-S) BAU/ml	40.9	<0.4	<0.4	<0.4	<0.4	<0.4	<0.4	<0.4	<0.4	19.18

Patient	Test	Observation period, months									
		1	2	5	6	7	8	9	10	11	12
ID_187	#4 (anti-N & S-RBD IgM)	-	-	-	-	-	-	-	g.z.	g.z.	-
	#3 (anti-S IgG)	+	-	-	-	-	-	+	+	+	g.z.
	#5 (total anti-N)	+	-	-	-	-	-	-	-	-	-
	#6 (total anti-S) BAU/ml	3.27	3.43	2.45	2.48	4.27	4.59	4.48	4.19	4.02	3.82

Pink rectangles represent months with positive result, green rectangles – months with a negative result and gray rectangles – indefinite (gray zone) result. N, nucleocapsid protein. S, spike protein. RBD, receptor binding domain. BAU/mL, binding antibody units per mL.
